# Analysis of failure causes and risk prediction of debridement, antibiotics, and implant retention (DAIR) for acute periprosthetic joint infection (PJI)

**DOI:** 10.3389/fcimb.2026.1621646

**Published:** 2026-01-23

**Authors:** Chaofan Zhang, Yubing Lu, Yiming Lin, Maocan Cai, Yishan Xin, Hongyan Li, Guochang Bai, Ye Yang, Zeyu Zhang, Yufeng Guo, Chengguo Huang, Wenbo Li, Yunzhi Lin, Wenming Zhang, Zida Huang, Xinyu Fang

**Affiliations:** 1 Department of Orthopaedic Surgery, National Regional Medical Center, Binhai Campus of the First Affiliated Hospital, Fujian Medical University, Fuzhou, China; 2 Department of Orthopaedic Surgery, the First Affiliated Hospital, Fujian Medical University, Fuzhou, China; 3 Fujian Provincial Institute of Orthopedics, the First Affiliated Hospital, Fujian Medical University, Fuzhou, China; 4 Fujian Orthopaedic Bone and Joint Disease and Sports Rehabilitation Clinical Medical Research, Fuzhou, Fujian, China; 5 Department of Orthopaedic Surgery, Changtai County Hospital, Zhangzhou, China; 6 Department of Orthopaedic Surgery, Pingnan County Hospital, Ningde, China; 7 Department of Stomatology, the First Affiliated Hospital, Fujian Medical University, Fuzhou, China; 8 Department of Stomatology, National Regional Medical Center, Binhai Campus of the First Affiliated Hospital, Fujian Medical University, Fuzhou, China

**Keywords:** Debridement, antibiotics and implant retention, antibiotics, debridement, nomogram, periprosthetic joint infection (PJA), predictive model, risk factors

## Abstract

**Objective:**

Debridement, antibiotics, and implant retention (DAIR) is the preferred treatment for acute periprosthetic joint infection (PJI), yet its failure rate remains high, and the influencing factors are not fully elucidated. This study aimed to investigate the causes of DAIR failure in acute PJI and construct a risk prediction model based on clinical characteristics, inflammatory markers, and microbiological data.

**Methods:**

A retrospective analysis was conducted on 90 patients with acute PJI treated at our medical center between January 2008 and April 2024. All patients underwent standard DAIR treatment and were categorized into success (n = 77) and failure (n = 13) groups based on outcomes. Demographic data, infection characteristics, laboratory markers, microbiological results, and surgical details were collected. Univariate and multivariate logistic regression analyses were performed to identify independent risk factors, and a nomogram prediction model was developed.

**Results:**

The overall success rate of DAIR was 85.6% (77/90). The failure group exhibited significantly higher rates of knee joint infection (84.6% vs. 50.6%, p=0.023), acute hematogenous infection (61.5% vs. 20.8%, p=0.005), preoperative peripheral White Blood Cell (WBC) (9.5×10^9/L vs. 8.2×10^9/L, p=0.043), CRP (79.6–4 mg/L vs. 42.4 mg/L, p<0.001), ESR (80.6 mm/h vs. 60.5 mm/h, p=0.002), synovial fluid WBC (35,300×10^6/L vs. 21,843×10^6/L, p=0.043), and synovial fluid polymorphonuclear leukocytes (PMNs) (91.7% vs. 83.8%, p<0.001) compared to the success group. Multivariate logistic regression identified acute hematogenous infection (OR 11.704, 95% CI 1.957–119.357, p=0.015), preoperative CRP (OR 1.022, 95% CI 1.009–1.040, p=0.003), synovial fluid PMN% (OR 1.196, 95% CI 1.039–1.454, p=0.039), and resistant pathogens (OR 0.107, 95% CI 0.010–0.665, p=0.032) as independent risk factors for DAIR failure. The nomogram model based on these factors demonstrated robust predictive performance.

**Conclusion:**

DAIR failure is closely associated with hematogenous infection, the intensity of inflammatory response, and the presence of resistant pathogens. The proposed risk prediction model may aid clinical decision-making and optimize patient selection for DAIR.

## Introduction

Periprosthetic joint infection (PJI) is one of the most severe complications following arthroplasty, with an incidence of 1–2% after primary procedures and 4–15% after revisions (Zimmerli, 2004). PJI not only leads to joint dysfunction and reduced quality of life but also imposes a significant economic burden (Kapadia et al., 2016). Diagnostic criteria for PJI include clinical signs (e.g., erythema, sinus tract), laboratory tests (e.g., elevated serum CRP, ESR), and synovial fluid analysis (e.g., leukocyte count, neutrophil percentage) (Parvizi et al., 2018). Despite advances in preventive measures, PJI remains a critical challenge in joint replacement.

Acute PJI typically refers to early postoperative infections or late hematogenous infections, characterized by sudden symptom onset and highly virulent pathogens (Tsukayama et al., 1996). Treatment options include DAIR, one- or two-stage revision, among others. DAIR is the preferred approach for acute PJI due to its minimal invasiveness, cost-effectiveness, and ability to retain the original implant (Byren et al., 2009). The procedure involves thorough debridement, exchange of modular components (e.g., polyethylene liner or femoral head), and combined local and systemic antibiotic therapy (Osmon et al., 2013).

Although DAIR is widely adopted, reported success rates vary significantly (26–92%) (Zhang et al., 2020). A meta-analysis indicated an overall success rate of only 46% (Kuiper, 2014). This variability may stem from differences in patient selection (e.g., symptom duration) (Kuiper et al., 2013), pathogen type (e.g., Staphylococcus aureus infections are associated with poorer outcomes) (Konigsberg et al., 2014), or surgical techniques (e.g., debridement adequacy) (Zhang et al., 2016). Additionally, factors such as advanced age (Buller et al., 2012), comorbidities (Ashkenazi et al., 2024), or CRP >150 mg/L (Wouthuyzen-Bakker et al., 2019a)have been identified as independent predictors of failure. Other researchers have identified biofilm formation time (>2 weeks), presence of sinus tract, and poor local soft tissue conditions as additional risk factors for DAIR failure (Azzam et al., 2010). Discrepancies in these findings highlight the need for standardized criteria to guide clinical decision-making.

This study aimed to retrospectively analyze acute PJI cases treated with DAIR at our institution, systematically evaluate failure causes (e.g., microbiological profiles, surgical techniques, host factors), and develop a risk prediction model incorporating inflammatory markers, clinical features, and pathogen characteristics to support individualized treatment strategies.

## Methods

### Study population and design

This was a single-center retrospective cohort study conducted at the First Affiliated Hospital of Fujian Medical University. This study was approved by the Institutional Review Board (approval no. MRCTA, ECFAH of FMU [2025] 007), and informed consent was obtained from all participants. The reporting of this study follows STROBE guidelines. Patients who underwent DAIR for acute PJI between 2008 and 2024 were retrospectively reviewed.

### Inclusion and exclusion criteria

***Inclusion criteria***: (i) Hip or knee arthroplasty patients diagnosed with acute PJI based on the 2011 Musculoskeletal Infection Society (MSIS) criteria (Parvizi et al., 2011), including early postoperative (Tsukayama type 2) and acute hematogenous (Tsukayama type 3) infections (Tsukayama et al., 1996). (ii) Patients treated with DAIR.

***Exclusion criteria***: (i) Infections occurring >3 months postoperatively or with symptoms lasting >3 months. (ii) Patients treated with one- or two-stage revision. (iii) Incomplete follow-up or lost-to-follow-up cases.

### Microbiology

Synovial fluid or pus was cultured in aerobic and anaerobic bottles, and tissue samples were processed for extended cultures (up to 14 days if necessary). Pathogen identification and resistance profiling were performed. Metagenomic next-generation sequencing (mNGS) was also conducted for culture-negative cases (BGI Group, Shenzhen, China). The results were normally available in 48 hours. In this study, resistant pathogens were specifically defined as microorganisms that demonstrated resistance to antibiotics in antimicrobial susceptibility testing.

### Surgical procedure

Our institution functions as a tertiary referral center for bone and joint infections in Fujian Province in the southeast of China. The majority of patients referred to our center had initially undergone arthroplasty procedures at other hospitals before being transferred for management of acute PJI. Upon confirming an acute PJI diagnosis, empirical antibiotic therapy was initiated immediately without awaiting microbiological culture results, followed by prompt DAIR procedures performed exclusively by a senior orthopedic surgeon (W. Zhang) using a standardized protocol.

The surgical procedure commenced with spinal anesthesia and thorough disinfection using iodine tincture followed by ethanol deiodination. Through the original incision, superficial debridement was performed before arthrotomy, during which synovial fluid was aspirated for both conventional cultures and mNGS. Following capsulotomy, a minimum of five periprosthetic tissue specimens were collected from distinct sites for microbiological analysis. The modular components were then exchanged, and all infected/necrotic tissues were systematically debrided. The surgical field underwent povidone-iodine immersion for 30 minutes followed by pulsed lavage with 3 liters of normal saline. After temporary wound closure and complete instrument exchange, the new components were implanted and the wound was closed in layers with drain placement for 48 hours, utilizing tourniquet control when necessary during knee procedures.

### Antibiotic therapy

Patients were initially treated with an empirical antibiotic regimen consisting of vancomycin (1.0 g intravenous infusion every 12 hours) combined with ceftazidime (2.0 g intravenous infusion every 8 hours) until culture or mNGS results became available. The antibiotic therapy was subsequently adjusted to target the specific pathogens based on antimicrobial susceptibility testing. In selected cases, infectious disease specialists were consulted for multidisciplinary evaluation, with routine monitoring of hepatic and renal function throughout the treatment course. The standard protocol involved 2 weeks of intravenous antibiotic administration followed by 4 weeks of oral antibiotic therapy.

### Data collection and statistical analysis

Comprehensive patient data were collected, including baseline characteristics and perioperative parameters (demographics, infection profiles, laboratory markers, microbiological findings, and surgical details). Treatment success was defined as: (1) resolution of clinical symptoms, (2) normalization of inflammatory markers (CRP, ESR, WBC), (3) absence of radiographic loosening, and (4) no requirement for lifelong suppressive antibiotics (minimum 1-year follow-up). Failure was defined as: (1) need for additional surgical interventions (repeat DAIR, debridement, one- or two-stage revision) or (2) indefinite antibiotic suppression. (Zhang et al., 2020) Univariate and multivariate logistic regression analyses were performed to compare variables between groups, followed by the construction of a nomogram prediction model.

### Development and validation of the nomogram predictive model

The construction of the predictive model followed a rigorous multi-step analytical process. Initial screening of potential predictors was performed through univariate logistic regression analysis, retaining variables with P-values ≤0.2 for further evaluation. These candidate variables were subsequently analyzed using least absolute shrinkage and selection operator (LASSO) logistic regression with 10-fold cross-validation to identify features with non-zero coefficients. The optimal lambda parameter was determined based on the one-standard-error rule (1se) from the minimum deviance. Variables selected through LASSO regression were then entered into a bidirectional stepwise multivariate logistic regression analysis, with statistical significance set at P<0.05 for both entry and retention in the final model. The resulting statistically significant predictors were incorporated into the nomogram construction. The developed nomogram underwent extensive validation procedures, including internal validation with 1000 bootstrap resamples to assess model stability. Calibration curves were generated to evaluate the agreement between predicted and observed outcomes. The model’s discriminative ability was examined through receiver operating characteristic (ROC) curve analysis, with calculation of the area under the curve (AUC), sensitivity, and specificity. Clinical utility was further assessed using decision curve analysis (DCA) to quantify net benefit across various probability thresholds. Additional validation was performed through 10-fold cross-validation.

All statistical analyses were conducted using specialized R packages: the “glmnet” package for LASSO regression, “rms” for nomogram construction and calibration plotting, “pROC” and “ggplot2” for ROC curve generation, and “ggDCA” for decision curve analysis. A two-tailed P-value <0.05 was considered statistically significant for all analyses.

### Statistical analysis

All data processing and statistical analyses were performed using R software (version 4.4.1). Continuous variables were presented as mean ± standard deviation when normally distributed, with between-group comparisons conducted using independent samples t-tests. Non-normally distributed continuous variables were expressed as median [P25, P75] and analyzed using the Wilcoxon rank-sum test. Categorical variables were summarized as counts (percentages), with between-group comparisons performed using chi-square tests or Fisher’s exact test when appropriate. Missing data were handled through multiple imputation using the “missRanger” package in R.

## Results

### Demographics

The study cohort comprised 90 patients who met the inclusion criteria for the final analysis. The population consisted of 45 male and 45 female participants. The mean patient age was 67.1 ± 11.5 years, with an average body mass index (BMI) of 25.5 ± 3.2 kg/m². Regarding infection sites, 40 cases (44.4%) developed following hip arthroplasty procedures, while 50 cases (55.6%) occurred after knee arthroplasty. Among the hip procedures, total hip arthroplasty (THA) accounted for 31 cases (77.5% of hip infections) and hemiarthroplasty for 9 cases (22.5%). For knee procedures, total knee arthroplasty (TKA) represented 41 cases (82.0% of knee infections) and unicompartmental knee arthroplasty (UKA) comprised 9 cases (18.0%). Based on the Tsukayama classification system, the cohort included 66 cases (73.3%) of early postoperative infection and 24 cases (26.7%) of acute hematogenous infection.

### Treatment outcomes

The study demonstrated an overall treatment success rate of 85.6% (77/90), with 77 successful cases and 13 failures. Baseline characteristics between groups showed no statistically significant differences in age, gender, BMI, Charlson comorbidity index (CCI), or presence of oozing (all p>0.05). (**Table 1**) Significant between-group differences were observed in both infection location and type (p=0.023 and p=0.002, respectively). The success group comprised 38 hip cases (95% success rate among total hip PJIs) and 39 knee cases (78% success rate among total knee PJIs), while the failure group included only 2 hip cases but 11 knee cases. This disparity suggests greater DAIR failure susceptibility in knee procedures. Among knee infections, success rates were comparable between TKA (78.0%; 32/41) and UKA (77.8%; 7/9). Regarding infection classification, the success group consisted of 61 early postoperative infections and 16 acute hematogenous infections, whereas the failure group contained 5 early infections versus 8 hematogenous cases. This distribution indicates substantially higher failure rates for acute hematogenous infections compared to early postoperative cases (33.3% vs 7.6% failure rate).

**Table 1:** Demographics of success and failure groups.

Variables	Success (N = 77)	Failure (N = 13)	P value
Age	67.0 ± 12.2	68.2 ± 7.0	0.619
Sex, n (%)			0.368
Male	40.0	5.0	
Female	37.0	8.0	
BMI	25.5 ± 3.1	25.2 ± 4.1	0.804
CCI	5.6 ± 3.9	6.2 ± 3.5	0.607
Oozing, n (%)			0.508
Yes	20 (26.0)	2 (15.4)	
No	57 (74.0)	11 (84.6)	
Infection site			0.023*
Hip	38 (49.4)	2 (15.4)	
Knee	39 (50.6)	11 (84.6)	
Infection type			
Tsukayama 2	61 (79.2)	5 (38.5)	0.005**
Tsukayama 3	16 (20.8)	8 (61.5)	
Follow-up (months)	66.1 ± 29.6	58.4 ± 36.1	0.478

(CCI, Charlson comorbidity index; BMI, body mass index).

* Significant at P < 0.05 level; ** Significant at P < 0.01 level.

**Table 2** presents the comparison of perioperative parameters between the two patient groups.

**Table 2:** Perioperative parameters of success and failure groups.

Variables	Success (N = 77)	Failure (N = 13)	P value
ASA	2.0 (2.0, 2.0)	2.0 (0.0, 2.0)	0.319
WBC (×10^9/L)	8.2 (5.9, 9.2)	9.5 (8.2, 15.1)	0.043
CRP (mg/L)	42.4 (16.7, 76.4)	79.6 (73.4,126.0)	< 0.001**
ESR (mm/h)	60.5 ± 33.1	80.6 ± 16.1	0.002
Synovial WBC (×10^6/L)	21,843.0 (5,580.0, 43,568.0)	35,300.0 (16,806.0, 70,851.0)	0.098
Synovial PMN (%)	83.8 (70.8, 90.1)	91.70 (89.8, 94.9)	< 0.001**

(ASA, American Society of Anesthesiologists Score; PMN, polymorphonuclear leukocytes. Data were presented as mean ± standard deviation or median [P25, P75]).

** Significant at P < 0.01 level.

The analysis of perioperative indicators showed no statistically significant differences in ASA classification between the success and failure groups. However, the failure group demonstrated significantly elevated levels across multiple inflammatory markers compared to the success group. Preoperative peripheral blood tests revealed higher white blood cell counts (9.5×10^9/L vs 8.2×10^9/L, p=0.043), CRP levels (79.6 mg/L vs 42.4 mg/L, p<0.001), and ESR values (80.6 mm/h vs 60.5 mm/h, p=0.002) in the failure group. Synovial fluid analysis similarly showed more pronounced inflammation in cases of DAIR failure, with significantly higher leukocyte counts (35,300×10^6/L vs 21,843×10^6/L, p=0.043) and polymorphonuclear neutrophil percentages (PMN) (91.7% vs 83.8%, p<0.001). These findings collectively indicate that both systemic and local inflammatory responses were more severe in patients who ultimately failed DAIR treatment, with CRP and PMN% showing particularly strong discriminatory value for predicting treatment outcomes.

### Microbiological findings

**Figure 1:**
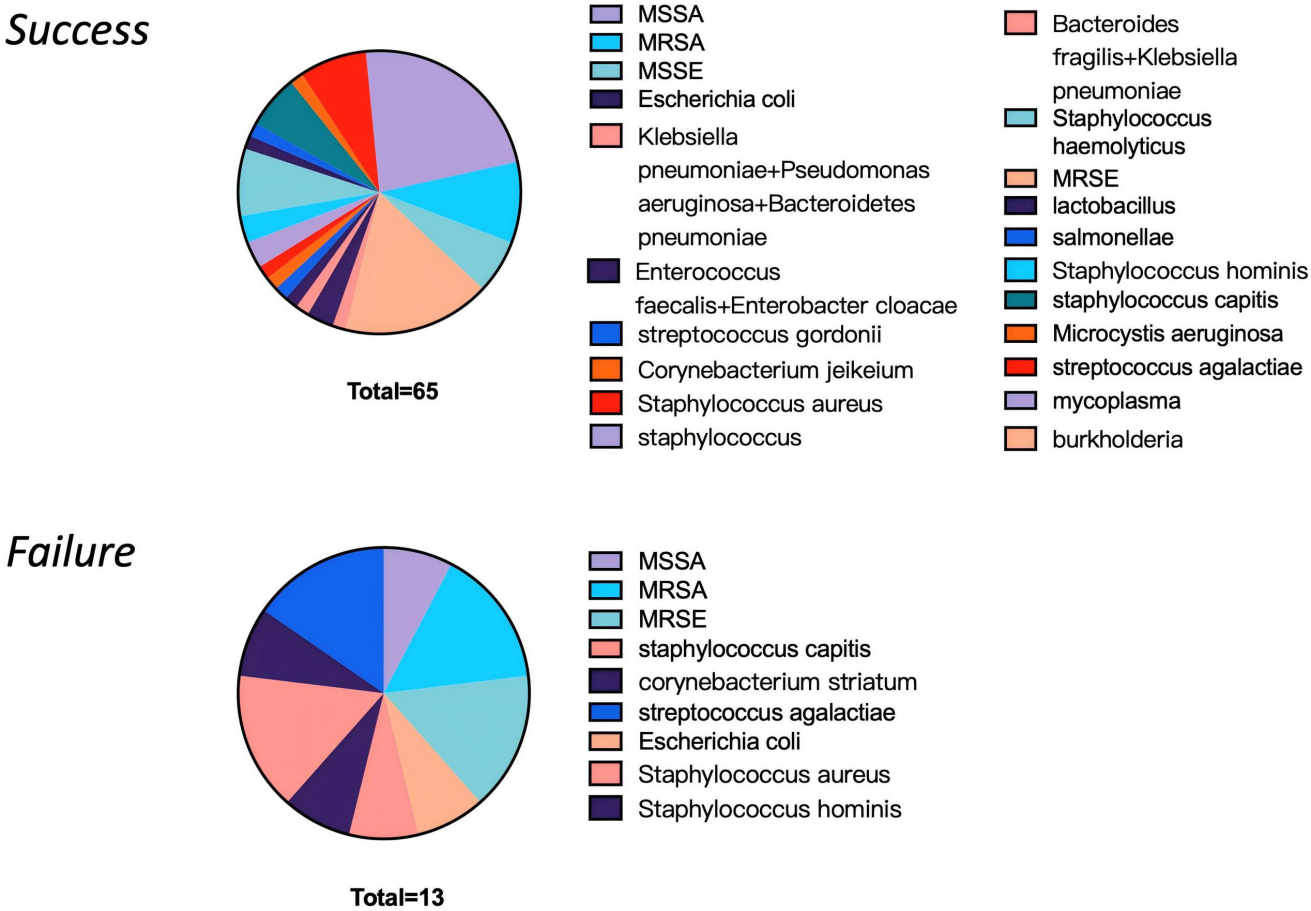
Microbiological identification results of the success and failure groups.

Microbial culture results were positive in 74 cases, yielding a culture positivity rate of 82.2% (74/90). Among the 16 culture-negative cases, 4 were subsequently identified as positive through mNGS testing. The pathogens identified in these four mNGS-positive cases were burkholderia, Microcystis aeruginosa, staphylococcus and Staphylococcus haemolyticus. **Figure 1** presents the comparative microbiological profiles between the two outcome groups. In the success group, microbial identification was achieved in 65 cases, while all patients in the failure group had confirmed microbiological results. (**Figure 1**)

The extended microbiological analysis revealed that several factors did not significantly influence surgical outcomes, including pathogen-negative status, presence of polymicrobial infection, and Gram-positive staining characteristics. However, a notable trend was observed regarding antimicrobial resistance patterns, with the failure group demonstrating a higher incidence of resistant pathogen infections compared to the success group, though this difference did not reach conventional statistical significance (p=0.106). (**Table 3**)

**Table 3:** Microbiological results of success and failure groups.

Variables	Success (N = 77)	Failure (N = 13)	P value
Pathogen status, n (%)			0.202
Positive	65 (84.4)	13 (100.0)	
Negative	12 (15.6)	0 (0.0)	
Polymicrobial infection, n (%)			
Yes	3 (3.9)	0 (0.0)	1.000
No	74 (96.1)	13 (100.0)	
Gram-staining, n (%)			0.613
Positive	70 (90.9)	11 (84.6)	
Negative	7 (9.1)	2 (15.4)	
Antimicrobial resistance patterns, n (%)			0.106
Resistant	29 (37.7)	8 (61.5)	
Sensitive	48 (62.3)	5 (38.5)	

### Univariate and multivariate regression analysis

The univariate regression analysis (**Table 4**) identified several potential risk factors for DAIR failure: infection site (hip/knee, p=0.036), infection type (early postoperative/hematogenous, p=0.004), preoperative peripheral WBC count (p=0.011), CRP level (p=0.003), ESR value (p=0.041), synovial fluid PMN% (p=0.006), duration of infection symptoms (p=0.062), and presence of resistant pathogens (p=0.114). To address potential multicollinearity among these variables, all factors with P ≤ 0.1 in the univariate analysis were included in the LASSO regression analysis. The LASSO regression with 10-fold cross-validation identified the optimal lambda value (logλ≈-3.5, **Supplementary Figure 1**), selecting four significant variables for the final multivariate model: infection type, preoperative CRP, synovial fluid PMN%, and microbial resistance. The multivariate logistic regression analysis (**Table 5**) confirmed these as independent predictive factors, with hematogenous infection (OR = 11.704, 95% CI: 1.957-119.357) and preoperative CRP (OR = 1.022, 95% CI: 1.009-1.040) demonstrating the strongest predictive value for DAIR failure. (**Table 5**)

**Table 4:** The univariate regression analysis of the success and failure groups.

Variable	β	SE	z	OR (95%CI)	P value
Sex					
Male	0.0			reference	
Female	0.5	0.6	0.9	1.7 (0.5, 6.2)	0.372
Infection site					
Hip	0.0			reference	
Knee	1.7	0.8	2.1	5.4 (1.3, 36.1)	0.036*
BMI	-0.0	0.1	-0.3	1.0 (0.8, 1.2)	0.755
Oozing					
Yes	0.0			reference	
No	0.7	0.8	0.8	1.9 (0.5, 13.2)	0.418
Type					
Tsukayama 2	0.0			reference	
Tsukayama 3	1.8	0.6	2.8	6.1 (1.8, 22.7)	0.004**
ASA	-0.3	0.3	-1.0	0.7 (0.4, 1.3)	0.303
WBC	0.2	0.1	2.5	1.2 (1.0, 1.4)	0.011*
CRP	0.0	0.0	3.0	1.0 (1.0, 1.0)	0.003**
ESR	0.0	0.0	2.0	1.0(1.0, 1.0)	0.041*
Synovial WBC	0.0	0.0	0.9	1.0 (1.0, 1.0)	0.373
Synovial PMN	0.2	0.1	2.7	1.2 (1.1, 1.4)	0.006**
Symptom duration	0.0	0.0	1.9	1.0 (1.0, 1.1)	0.062*
Pathogen status					
Positive	0.0			reference	
Negative	-16.9	1882.9	-0.0	0.0 (0.0, NA)	0.993
Polymicrobial					
Yes	0.0			reference	
No	14.8	1385.4	0.0	2749563.4 (0.0, NA)	0.991
Gram-staining					
Positive	0.0			reference	
Negative	0.6	0.9	0.7	1.818 (0.3, 8.8)	0.489
Antimicrobial resistance					
Resistant	0.0			reference	
Sensitive	-1.0	0.6	-1.6	0.378 (0.1, 1.2)	0.114*

(PMN, polymorphonuclear leukocytes).

* Significant at P < 0.05 level; ** Significant at P < 0.01 level.

**Table 5:** The multivariate regression analysis of the success and failure groups.

Variable	β	SE	z	OR (95%CI)	P value
Type					
Tsukayama 2	0.0			reference	
Tsukayama 3	2.5	1.0	2.4	11.7 (2.0,119.4)	0.015
CRP	0.0	0.0	2.9	1.0 (1.0,1.0)	0.003
Synovial PMN	0.2	0.1	2.1	1.2 (1.0,1.5)	0.039
Antimicrobial resistance					
Resistant	0.0			reference	
Sensitive	-2.2	1.0	-2.1	0.1 (0.0,0.7)	0.032

(PMN, polymorphonuclear leukocytes).

### ROC curve analysis of inflammatory markers

**Figure 2:**
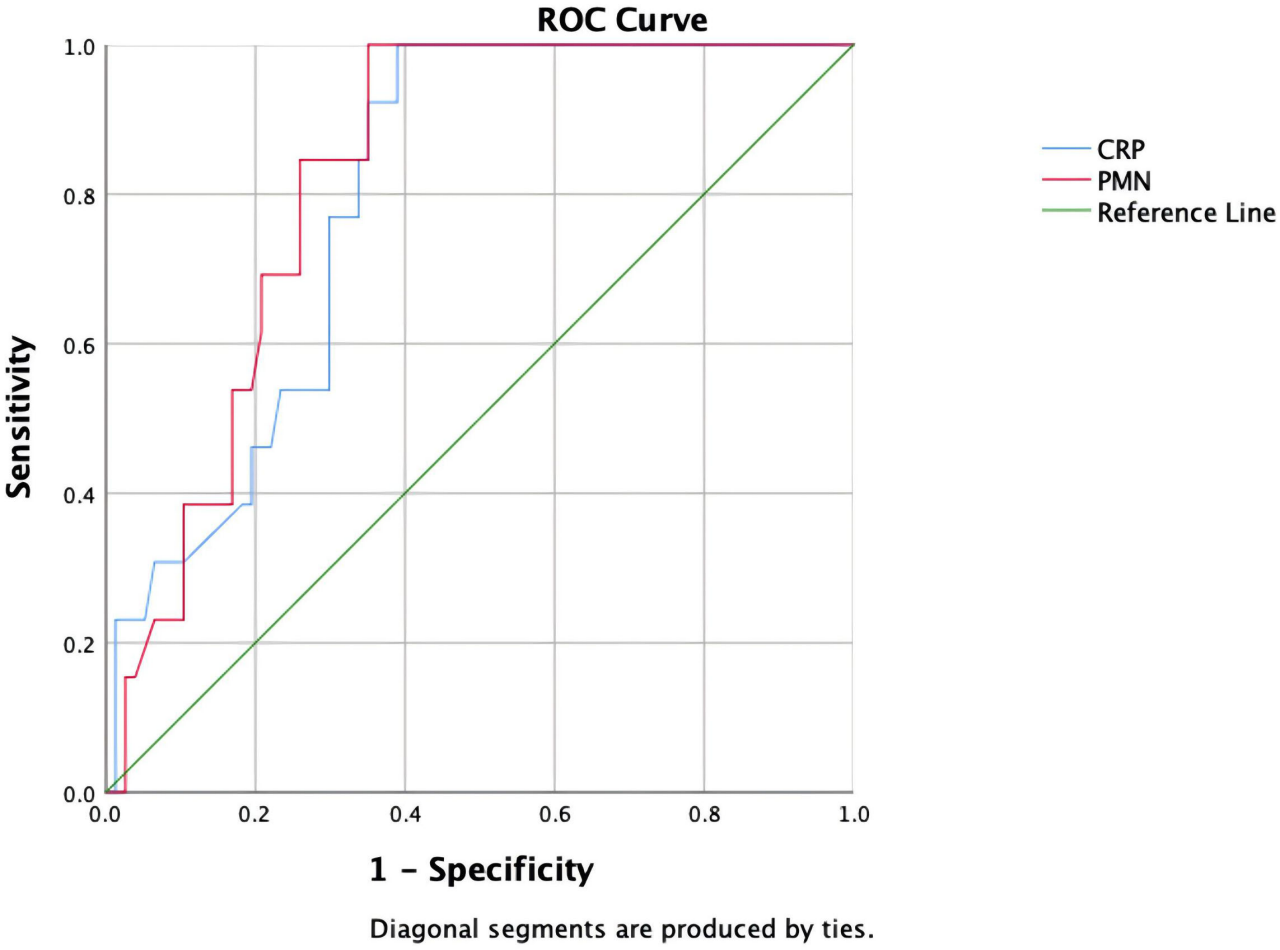
ROC curves for CRP and synovial fluid PMN%.

The predictive performance of inflammatory markers was further evaluated through ROC curve analysis (**Figure 2**). CRP demonstrated significant discriminative ability for DAIR treatment failure with an AUC of 0.797 (95% CI: 0.692-0.902). Synovial fluid PMN% showed superior predictive accuracy compared to CRP, achieving an AUC of 0.825 (95% CI: 0.726-0.924). Using the Youden index, optimal cutoff values were determined to maximize predictive performance. For CRP, the threshold of 63.3 mg/L provided perfect sensitivity (100%) while maintaining 61% specificity. Similarly, a PMN% cutoff of 87.4% achieved 100% sensitivity with 64.9% specificity. These results suggest that while both markers show excellent sensitivity for identifying potential treatment failures, PMN% may offer marginally better overall performance in clinical decision-making.

### Development and validation of the predictive model

**Figure 3:**
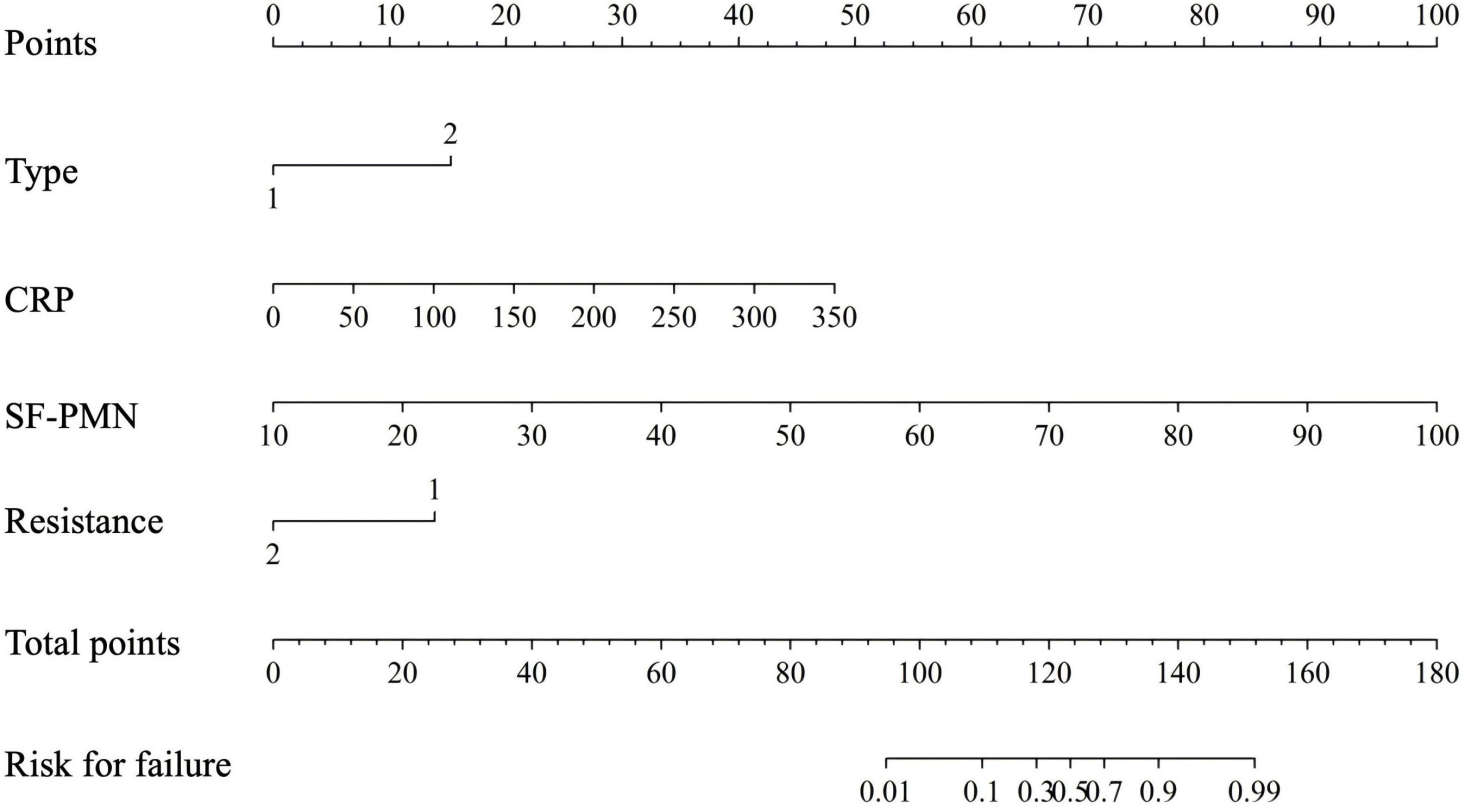
Nomogram for predicting the risk of treatment failure based on multivariate logistic regression analysis. For clinical application: (1) Locate the patient’s clinical values (e.g., infection type, preoperative CRP) on the corresponding variable axes; (2) Project upward to determine the points for each parameter; (3) Sum all points to obtain the total score (range: 0-180); (4) Project downward from the total score to the “Risk of Treatment Failure” axis to determine the individualized probability of DAIR failure. (For infection type, ‘1’ means ‘Tsukayama 2’ and ‘2’ means ‘Tsukayama 3’. For resistance, ‘1’ means ‘yes’ and ‘2’ means ‘no’. SF-PMN: synovial fluid polymorphonuclear leukocytes).

The predictive nomogram (**Figure 3**) was developed incorporating the four independent risk factors identified in the multivariate analysis, with a total possible score ranging from 0 to 180 points. The nomogram displays individual variable scores on the top scale, where clinical values can be converted to points by drawing vertical lines. The sum of these points corresponds to a total score on the bottom scale, which can then be projected downward to estimate the probability of treatment failure. The model’s predictive performance was rigorously evaluated through multiple validation methods. ROC curve analysis demonstrated strong discriminative ability, while calibration plots showed excellent agreement between predicted and observed outcomes. Decision curve analysis further confirmed the model’s clinical utility across various probability thresholds (**Supplementary Figures 2–4**).

## Discussion

Despite being widely recommended as the first-line treatment for acute PJI, DAIR continues to demonstrate substantial failure rates (14.4% in our center, though comparatively lower than many institutions). This persistent challenge reflects several critical issues in current clinical practice. The complex pathophysiology of acute PJI involves multiple factors including biofilm formation and dysregulated host immune responses (Zimmerli, 2004). Furthermore, existing treatment strategies show marked variability in reported success rates (30-90%) (Zhang et al., 2020), likely attributable to inconsistencies in patient selection criteria, surgical techniques (Kuiper, 2014), and antibiotic protocols (Tornero et al., 2015). These findings collectively underscore the urgent need for standardized, multidimensional assessment systems to optimize treatment strategies.

Our systematic analysis of 90 acute PJI cases yielded several significant findings. Multivariate analysis identified infection type (p=0.004), preoperative CRP (p=0.003), PMN% (p=0.039), and resistant pathogens (p=0.032) as independent risk factors for DAIR failure. These results align with clinical observations, suggesting caution when considering DAIR for knee arthroplasty infections with elevated inflammatory markers or resistant organisms. ROC analysis established optimal predictive thresholds for CRP (>63.3 mg/L) and PMN% (>87.4%), notably lower than previous benchmarks (150 mg/L) (Parvizi et al., 2018), indicating the need for stricter surgical indications. Our novel nomogram model integrates anatomical, microbiological, and host factors, offering distinct advantages over traditional KLIC scores (Tornero et al., 2015). First, it incorporates quantifiable inflammatory markers feasible for widespread clinical use. Second, decision curve analysis confirms its clinical utility. These advances enable personalized treatment approaches, particularly for high-risk patients (nomogram score ≥150) who may benefit from extended antibiotics or early revision.

Consistent with most published studies, our findings confirm that DAIR for acute PJI continues to demonstrate significant failure rates. This phenomenon appears multifactorial in origin, with several key mechanistic pathways identified. Firstly, the rapid formation of bacterial biofilms by common PJI pathogens (particularly Staphylococcus aureus and coagulase-negative staphylococci) on prosthetic surfaces creates substantial antibiotic penetration barriers (Urish et al., 2014). While our study did not directly assess biofilm formation, the significant association between prolonged symptom duration and treatment failure in our univariate analysis strongly supports the critical importance of the biofilm formation timeline (Wouthuyzen-Bakker et al., 2019a). This biological reality underscores the narrow therapeutic window for successful DAIR intervention. Secondly, anatomical considerations further complicate treatment outcomes. The markedly higher failure rates observed in knee versus hip PJI likely reflect the knee’s more complex synovial architecture, which poses greater challenges for complete surgical debridement (Lora-Tamayo et al., 2013). Additionally, the polyethylene liners in knee prostheses may harbor occult infection foci, while the inherent mobility of hip components could enhance local antibiotic distribution (Byren et al., 2009). These biomechanical differences have important implications for case selection and surgical planning. Thirdly, our data also reveal significant host immune factors contributing to DAIR failure. The substantially elevated CRP and synovial PMN% levels in the failure group suggest that excessive inflammatory responses may paradoxically both exacerbate tissue damage and impair pathogen clearance (Sabry et al., 2014). This finding supports the “infection-inflammation vicious cycle” hypothesis proposed by Tornero et al. (2015), though our identified CRP threshold (63.3 mg/L) differs from the 150 mg/L cutoff reported by Wouthuyzen-Bakker et al. (2019b), potentially reflecting population variations or methodological differences in threshold determination.

Our investigation revealed two further significant determinants of treatment failure. First, infection type substantially influenced outcomes according to the Tsukayama classification (Tsukayama et al., 1996). While DAIR remains recommended for both early postoperative and acute hematogenous infections, we observed markedly higher failure rates in hematogenous cases. This likely reflects the disseminated nature of hematogenous spread, which facilitates multifocal biofilm colonization (Lora-Tamayo et al., 2013). An alternative explanation suggests some presumed acute hematogenous infections may actually represent exacerbations of preexisting chronic low-grade infections, with the acute presentation triggered by secondary factors - a scenario particularly challenging for DAIR success. Second, antimicrobial resistance emerged as a critical risk factor, corroborating Byren et al.’s findings (Byren et al., 2009). Resistant pathogens not only substantially increase recurrence risk but also severely limit effective antibiotic options, creating a therapeutic dilemma. This dual challenge of enhanced microbial persistence and constrained pharmacological arsenal underscores the need for comprehensive microbiological assessment and tailored antibiotic strategies when considering DAIR for resistant infections.

The microbiological analysis in this study provides important insights into DAIR failure. From a diagnostic standpoint, applying mNGS to 16 culture-negative cases successfully identified pathogens in 4 instances, allowing for targeted antibiotic adjustments. This underscores the value of mNGS as a complementary diagnostic tool for acute PJI, particularly in improving pathogen detection after empirical treatment (Huang et al., 2020). Furthermore, our analysis revealed a notable pattern: while all treatment failures had a confirmed pathogen (primarily Staphylococcus species, indicating a relatively narrow spectrum), the success group exhibited a more diverse pathogen profile despite including culture-negative cases (**Figure 1**). This suggests that failure may be associated with infections driven by specific, often resistant, pathogens. Consequently, preoperative assessment should extend beyond merely detecting a pathogen to critically evaluating the risk implied by the pathogen’s identity and resistance profile.

Our newly developed nomogram represents a significant advancement over traditional scoring systems like the KLIC score (Tornero et al., 2015), offering two key clinical advantages: First, the model incorporates quantifiable inflammatory markers (CRP and PMN%) that are routinely available through standard laboratory testing. This practical feature facilitates widespread adoption across healthcare facilities of varying resource levels, enabling rapid risk stratification without requiring specialized assays or complex calculations. The immediate accessibility of these biomarkers allows for real-time clinical decision-making during critical perioperative periods. Second, the nomogram enables truly personalized therapeutic strategies. For high-risk patients (nomogram score ≥150), clinicians can consider more aggressive approaches including 1) early revision arthroplasty, 2) extended antibiotic regimens (Byren et al., 2009), or 3) adjuvant biofilm-disrupting agents (Tande and Patel, 2014). This risk-adapted paradigm represents a substantial improvement over the binary recommendations of the KLIC system, aligning with the growing consensus for individualized PJI management advocated by Parvizi et al. (2013). The model’s ability to discriminate subtle gradations of risk allows for optimized resource allocation - reserving intensive therapies for those most likely to benefit while avoiding overtreatment of lower-risk cases.

A key strength of this study is the development and validation of a clinically applicable nomogram that integrates acute-phase-specific inflammatory markers (synovial fluid PMN% and preoperative CRP) with the presence of resistant pathogens. This model offers two distinct advances: it provides optimized early-warning thresholds (CRP >63.3 mg/L, PMN% >87.4%) that are more sensitive than traditional benchmarks, and it generates a personalized estimate of failure risk. This facilitates more precise, individualized decision-making regarding the surgical approach, such as whether to proceed with DAIR or consider direct revision.

Several important limitations must also be acknowledged in interpreting our findings. Firstly, the retrospective study design introduces inherent biases, particularly regarding surgical technique variability. Factors such as debridement thoroughness and antibiotic-loaded cement utilization were not standardized across cases, potentially influencing treatment outcomes in ways our analysis could not fully account for. Secondly, our microbiological analysis, while comprehensive for clinical purposes, lacked molecular depth. We did not perform virulence factor profiling or host genetic polymorphism analysis, which could have provided mechanistic insights into individual treatment failures. Such molecular characterization might explain outlier cases where clinical predictors and outcomes diverged. Third, all patients in this cohort received a uniform 12-week antibiotic regimen, which minimized bias related to treatment duration variation. However, this differs from practices at some institutions. For instance, the multi-center DATIPO trial included units using only 6 weeks of antibiotics (Bernard et al., 2021). Furthermore, the newly released International Consensus Meeting for Musculoskeletal Infection (ICM) 2025 guidelines recommend a total antibiotic course of 3 months following DAIR (Cashman et al., 2025). Therefore, further guideline development is needed to standardize antibiotic protocols, which would enhance the comparability of outcomes across different studies. Fourth, the mean BMI of patients in this and other similar studies from East Asia is notably lower than that typically reported in Western cohorts (Espíndola et al., 2022) (Chang et al., 2022). This demographic difference must be considered when interpreting the generalizability of the treatment outcomes. Future prospective studies involving more diverse populations are warranted to further validate these findings. Most critically, the nomogram currently lacks external validation. While internal validation through bootstrap resampling showed promising results, the model’s generalizability remains unproven.

## Conclusion

DAIR treatment for acute PJI continues to demonstrate a certain failure rate. Treatment failure shows strong associations with hematogenous infection, intensity of inflammatory response, and presence of drug-resistant microorganisms. The risk prediction model developed in this study may serve as a valuable clinical decision-support tool to optimize patient selection.

## Data Availability

The raw data supporting the conclusions of this article will be made available by the authors, without undue reservation.
