# Supplementary material for: Analysis of failure causes and risk prediction of debridement, antibiotics, and implant retention (DAIR) for acute periprosthetic joint infection (PJI)
Source: Front Cell Infect Microbiol. 2026 Jan 23;16:1621646. doi: 10.3389/fcimb.2026.1621646 (PMC12876192; doi:10.3389/fcimb.2026.1621646)
Supplement: Supplementary file 1 [file Table1.docx]

**Supplemental Materials**


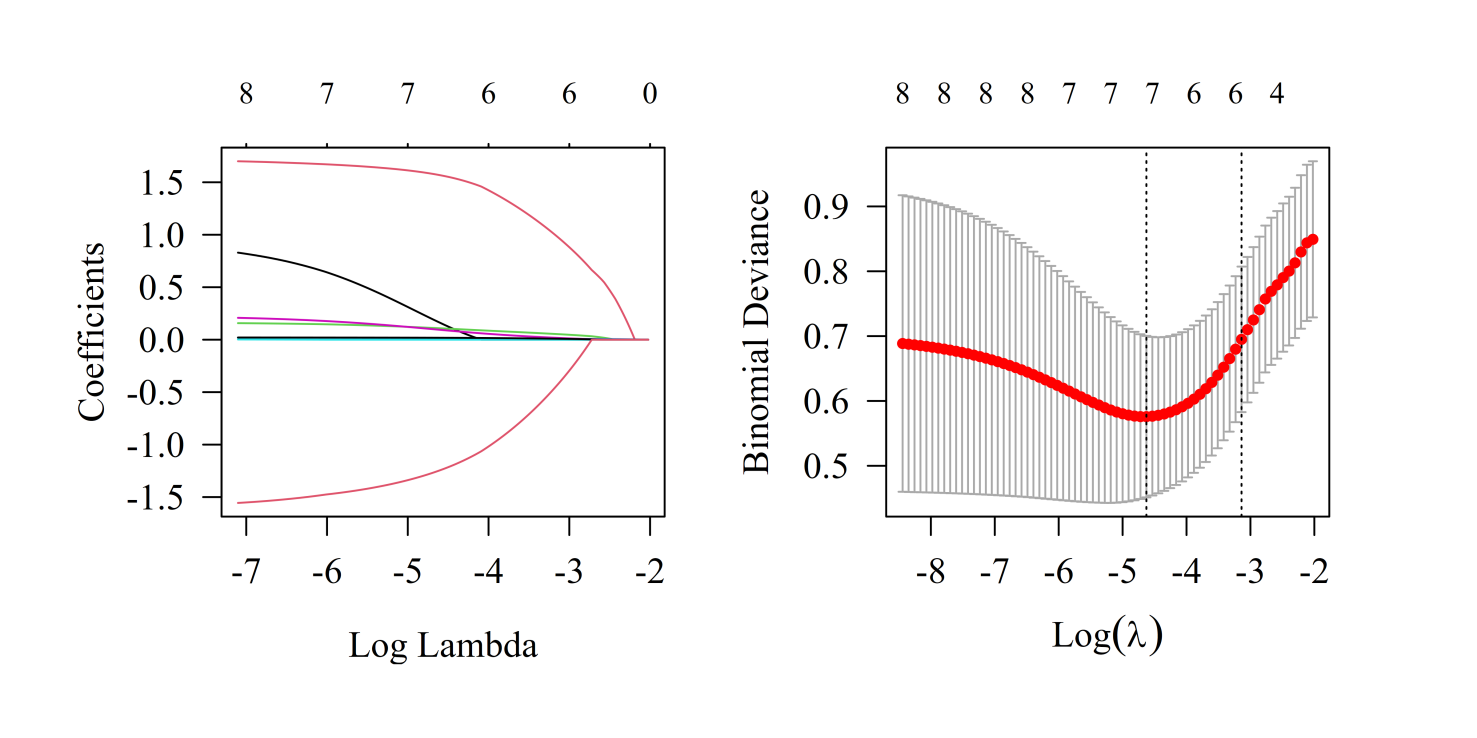


**Figure 1. Results of LASSO regression analysis.** (Left) Coefficient profiles of the 8 potential risk factors (see Table 1) analyzed by LASSO regression. (Right) Ten-fold cross-validation curve for selecting the optimal lambda value (1 se), which identified 4 significant variables for inclusion in the subsequent multivariate regression analysis.

The corresponding ROC curve, calibration curve, and decision curve were constructed for the nomogram. The ROC analysis demonstrated excellent predictive performance with an AUC of 0.923 (95% CI: 0.841-1.000), sensitivity of 0.846 (0.650, 1.000), and specificity of 0.896 (0.828, 0.964) (**Figure 2**). Furthermore, the model underwent additional validation through 10-fold cross-validation, yielding an AUC of 0.880 (0.776, 0.984) with maintained high sensitivity (0.846; 0.650-1.000) and specificity (0.844; 0.763-0.925), confirming its robust predictive capability.


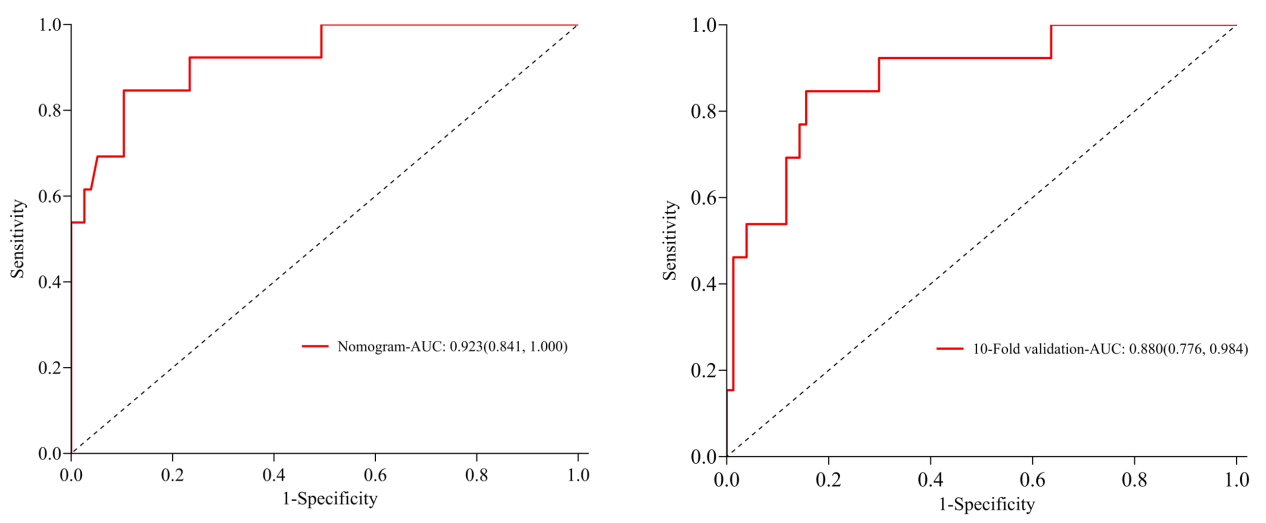


**Figure 2. ROC curves of the nomogram model.** (Left) ROC curve from bootstrap validation showing an AUC of 0.923 (0.841-1.000). (Right) ROC curve from 10-fold cross-validation demonstrating an AUC of 0.880 (0.776-0.984).

Bootstrap validation with 1000 resamples demonstrated excellent agreement between predicted probabilities and observed outcomes, as shown in the calibration curve. The model achieved a mean absolute error of 0.025, indicating high predictive accuracy.


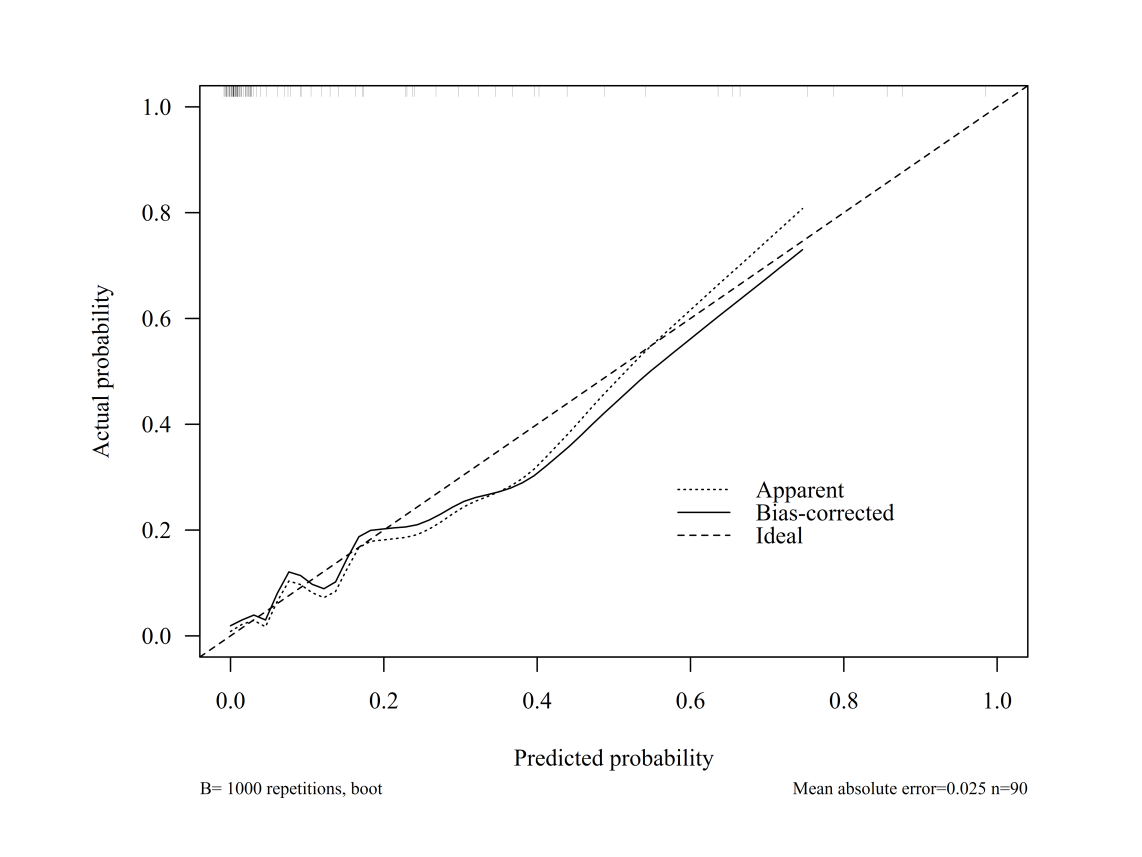


**Figure 3. Calibration curve of the nomogram model.** The x-axis represents predicted probability, while the y-axis shows actual probability. The plot includes three reference lines: "Ideal" (perfect prediction line), "Apparent" (observed prediction probability from the original data), and "Bias-corrected" (bootstrap-corrected probability from 1000 resamples), demonstrating the model's performance in aligning predicted and observed outcomes.

The decision curve analysis demonstrated consistent clinical net benefit across the threshold probability range of 0.1-0.9, indicating broad potential applicability of this predictive model in clinical practice. (**Figure 4**)


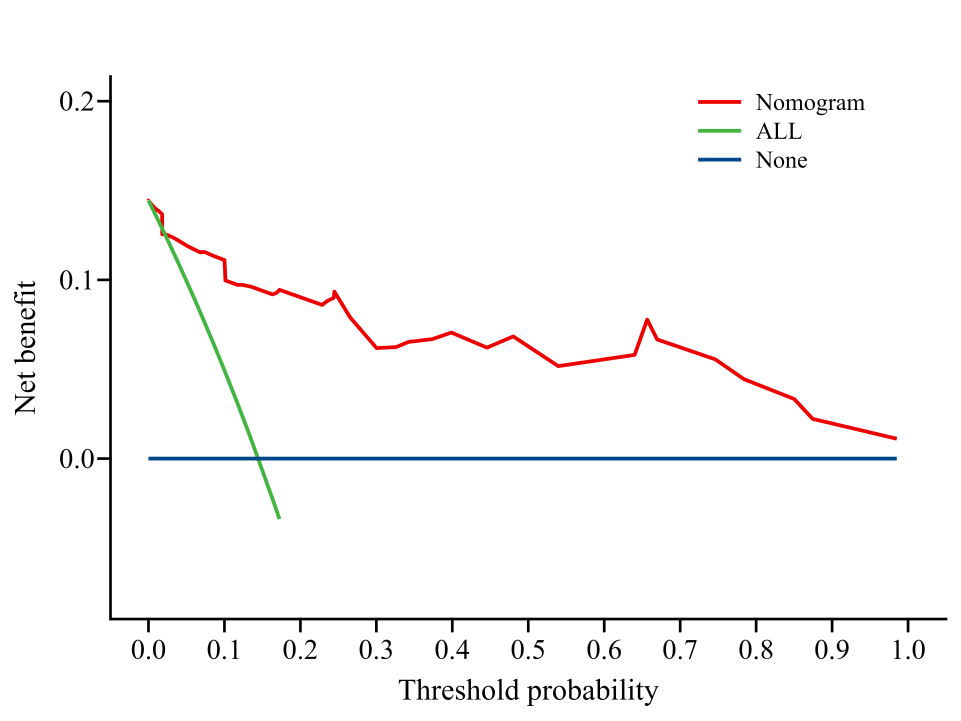


**Figure 4. Decision curve analysis (DCA) of the nomogram model**. The curves depict the expected net benefit of using this nomogram to predict treatment failure risk: the red solid line represents net benefit when intervening based on nomogram predictions across various threshold probabilities; the blue solid line indicates the "treat none" strategy; and the green solid line shows the "treat all" strategy's net clinical benefit.
